# Precision‐Optimised Post‐Stroke Prognoses

**DOI:** 10.1002/acn3.70077

**Published:** 2025-06-12

**Authors:** Thomas M. H. Hope, Howard Bowman, Rachel M. Bruce, Alex P. Leff, Cathy J. Price

**Affiliations:** ^1^ Department of Imaging Neuroscience, Institute of Neurology University College London London UK; ^2^ Department of Psychology and Social Sciences John Cabot University Rome Italy; ^3^ Department of Brain Repair and Rehabilitation UCL Queen Square Institute of Neurology London UK

**Keywords:** cognition, confidence, language, lesions, machine learning, stroke

## Abstract

**Background:**

Current medicine cannot confidently predict who will recover from post‐stroke impairments. Researchers have sought to bridge this gap by treating the post‐stroke prognostic problem as a machine learning problem, reporting prediction error metrics across samples of patients whose outcomes are known. This approach effectively shares prediction error equally among the patients, which is contrary to the long‐held clinical intuition that some patients' outcomes are more predictable than other patients' outcomes. Here, we test that intuition empirically, by asking whether those ‘more predictable’ patients can be identified before their outcomes are known.

**Methods:**

Drawing on lesion location and demographic data, we use ensemble classifiers to predict the presence of a variety of different language impairments in a large sample of stroke patients. We tune these models to maximise their Positive Predictive Value (or precision): that is, the probability that patients assigned to a class are really members of that class. We test whether those tuned models have high precision on independent data.

**Results:**

Precision‐tuned models might only classify a subset of patients, but for that reduced set, the classifications are very likely to be correct: typically > 90% and sometimes > 95%. Small reductions of target precision could rapidly raise the proportion of patients for whom ‘high enough precision’ predictions can be made.

**Conclusions:**

High precision prognoses are possible when predicting language outcomes after stroke. Providing such predictions for subsets of patients might be a reasonable intermediate step on the way to providing them for all.

## 1 Introduction

Stroke is the leading cause of overall disease burden in the world [1], and those who survive the initial insult often suffer from cognitive impairments. Language impairments (aphasia) occur in around 1/3 of stroke patients, and can be especially distressing [2]; these patients want to know whether and when they might recover [3]. Recently, many researchers have sought to fill this gap by learning associations between putative prognostic factors and outcomes, for patients whose outcomes are known, and generalising those models to predict outcomes for new patients (e.g., [4, 5, 6, 7]). The quality of these models is then typically reported via some measure of sample‐wide (or test‐set‐wide) prediction error, with smaller/fewer errors implying better predictions. This approach assumes that prediction error is distributed equally across the sample, implying that we cannot predict in advance the prognostic error associated with a specific individual. Though sensibly conservative, this assumption is contrary to the long‐held intuition that some patients' outcomes are more predictable than others' outcomes. If true, that intuition could allow for incremental solutions to the post‐stroke prognostic problm, whereby confident prognoses can be predicted for some patients, even before they can be made for all. Moreover, any reliable distinction between patients whose outcomes can versus cannot be predicted with high confidence, can potentially tell us something new about how the language system recovers after stroke. Here, we test this intuition empirically, demonstrating the feasibility of identifying ‘more predictable’ patients without knowing their outcome scores in advance—and thereby introducing a method that can predict that subset of patients' outcomes with high confidence, after their stroke.

Different medical domains will define ‘high confidence’ differently. When a dermatologist examines a mole on a patient's skin, for example, the riskiest mistake is to dismiss a potentially fatal melanoma as something more benign. It is therefore imperative that most or all patients who are *cleared* of melanoma, actually do not have one. In other words, the Negative Predictive Value (NPV) for the melanoma classification must not fall below some fixed, and presumably very high threshold: perhaps approaching 100%. To ensure this, we might be prepared to accept a relatively high rate of false positives, because the cost of those errors is wasted resources (unnecessary biopsies) rather than lost lives. This implies a lower Positive Predictive Value (PPV): the proportion of positive classifications or diagnoses that prove to be correct. PPV is also known as *precision*. In this sense, practical melanoma‐detection models will likely operate under asymmetrical constraints on PPV and NPV.

Practical post‐stroke prognostic models might also operate with asymmetrical constraints. First, patients might want to be told that they are expected to recover even if the confidence in that prediction is not especially high. However, this should still be balanced against the potential disappointment if the prediction is wrong, potentially compounding their distress and even delaying the arrangement of support services that are necessary for long‐term aphasia. And the negative prediction—that they will not recover—might never be wanted no matter how high its confidence. Receiving this kind of bad news can cause its own harm to patients and also risks becoming a self‐fulfilling prophecy if it discourages patients from engaging with rehabilitation [8]. But it is sometimes evidently necessary to deliver bad news in many areas of medicine, and stroke is no exception.

Identifying such patients early makes it possible to manage their expectations, whilst employing mechanisms to preserve hope. This can include focusing on compensatory strategies/interventions and factors that can be modified, as well as highlighting support available [9, 10]. In the case of aphasia, this could include information‐giving, training in functional strategies, provision of alternative and augmentative communication, psychological support, and potentially putting long‐term care into place. All whilst devoting targeted research into new impairment‐based interventions for this group. Clinicians will naturally want to take the most optimistic stance possible on post‐stroke recovery, so might only be prepared to deliver negative prognoses if their confidence was extremely high (and perhaps not even then). In other words, the precision required when predicting that patients will not recover (a negative prediction), might be much higher than that required when predicting that patients will recover (a positive prediction). This is analogous to the imbalance for melanoma detection.

Stroke prognoses differ from dermatological diagnoses in another important respect. When judging whether a patient has melanoma, a dermatologist must either refer them for further tests or discharge them: they cannot simply refuse to decide either way. But it is currently permissible—even advisable—to refuse to predict individually specific post‐stroke prognoses, when clinicians doubt that those predictions can be made with confidence [11]. This context presents an opportunity to build on the long‐held clinical intuition that some patients' outcomes are more predictable than others. Even if the ultimate goal, of making confident predictions for all patients, still remains out of reach, perhaps we can still make confident predictions for some?

Here, we show that this is indeed possible, at least when predicting language outcomes after stroke. Using only standard model calibration methods and a simple classification model, we can tune the way we classify patients' outcomes so that those classifications are only made when their confidence is high. Our aim in this work was to measure whether we could make such high precision predictions reliably—or more formally, to measure whether this precision‐tuning process would generalise out of sample in this domain. Our results show that it does generalise well out of sample.

## 2 Methods

**FIGURE 1:**
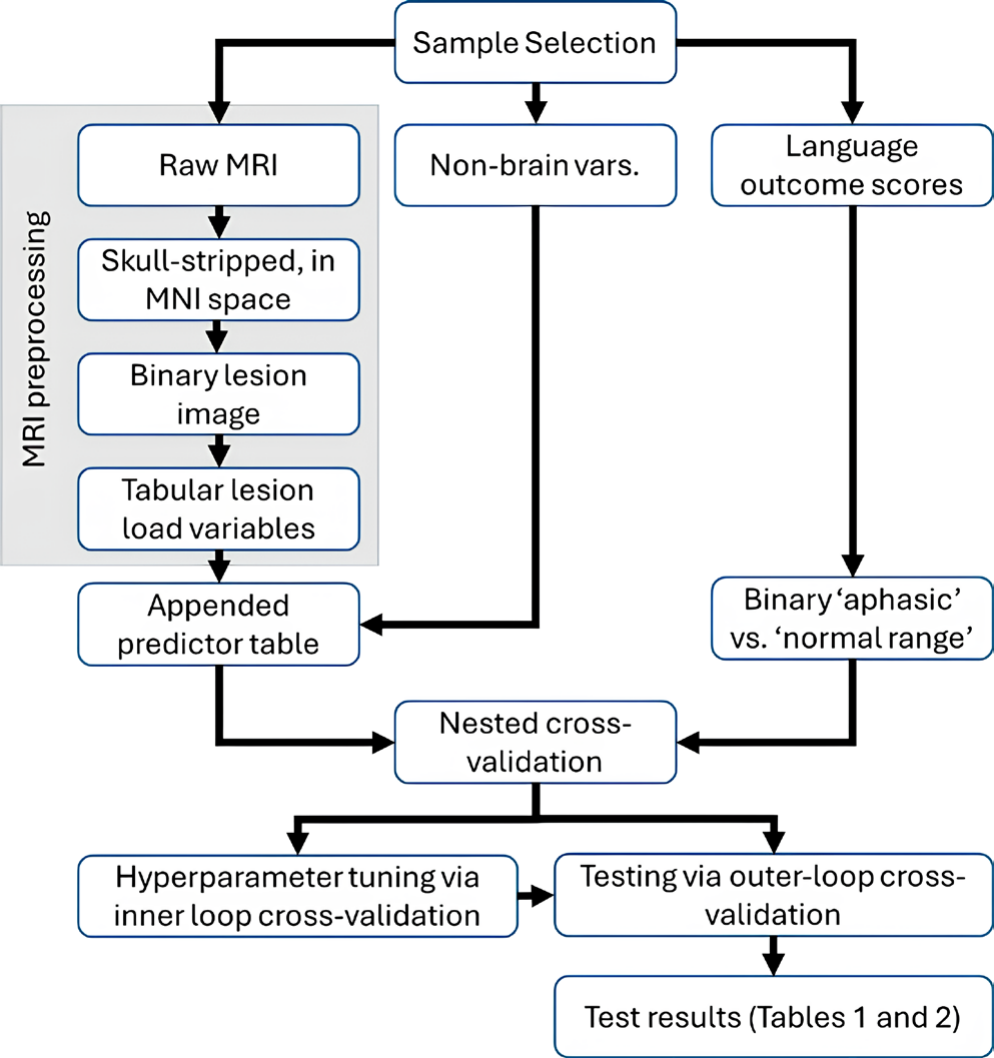
A schematic illustration of the data pre‐processing and analysis procedure. After selecting the sample, we extract MRI data, non‐brain demographic variables, and language outcome scores for the selected patients. Tables 1 and 2 report the results of the outer loop cross validation, emphasising those patients for whom ‘high precision’ classifications were made.

Our analysis proceeds from raw data, through several pre‐processing steps, to a model training and testing phase. A schematic of the procedure is displayed in Figure 1, which is followed by a more detailed explanation of the key steps.

### 2.1 Data

Our patient data are drawn from the Predicting Language Outcome and Recovery After Stroke (PLORAS) dataset [12], which associates more than 1500 stroke survivors with: (a) clinical and demographic data; (b) high resolution, T1‐weighted structural MRI; and (c) scores from the Comprehensive Aphasia Test (CAT) [13]: a standardisoverrepresentbehavioural tasks, designed to assess the severity of participants' language and cognitive impairments. Data were acquired primarily but not exclusively in the chronic phase, ranging from 2 months to > 10 years post‐stroke. We have described this dataset in detail elsewhere [12]; here, we repeat only those elements that are salient to the current analysis.

The CAT defines 34 task scores, including 29 that refer to language skills, and 5 that refer to non‐linguistic, general cognitive skills [13]. Here, we present analyses of 8 language summary scores, which represent the main language skills including: (a) verbal fluency; (b) auditory comprehension; (c) written comprehension; (d) repetition; (e) naming; (f) spoken scene description; (g) reading; and (h) writing. Detailed descriptions of these scores, and the tasks from which each score is derived, are provided in the manual for the CAT [13]. Scores are represented as *t*‐scores, defined relative to a distribution of scores for each task, acquired from 236 people with aphasia. Lower *t*‐scores imply worse task performance, and therefore more severe impairment in that task.

The T1‐weighted MRI scans were acquired using a variety of Siemens scanners since 2003—typically but not exclusively on the same day as, or within a few days of, the conduct of the behavioral assessment. We also acquired clinical and demographic data at the same time, such as the participants' age at stroke onset and their sex assigned at birth. Finally, a (large) subset of patients also completed our in‐house Aphasia Recovery and Therapy Questionnaire, asking them to rate the severity of their aphasic symptoms both soon after stroke and at the time of assessment. This provides the subjective self‐report data referred to below.

### 2.2 Sample Selection

The PLORAS dataset excludes patients who have had multiple strokes, or who suffer serious comorbid neurological conditions (such as Alzhemier's or Parkinson's disease). From this set, we selected all patients who had complete outcome scores data in at least one of our 8 domains of interest. Where patients had visited us more than once, we used only the data from the first visit. This resulted in a sample of 1367 patients, including 458 women and 909 men. This is a much more skewed ratio than would be expected in the wider stroke survivor population (e.g., [14]), and this difference could be important here because the incidence of post‐stroke aphasia is also thought to be slightly higher in women than men [15] although the severity of aphasia is reported to be worse in men than women [16]. So, we give this variable extra attention in our analyses. 1195 of the participants were right handed pre‐stroke, with the remainder either left handed or ambidextrous. 1223 were native English speakers. The average age at stroke was 56 years ± 13.3 years, and the average time post‐stroke at assessment was 46.3 months ± 56.8 months. The average lesion volume was 64.0 cm^3^ ± 82.7 cm^3^.

### 2.3 MRI Pre‐Processing

All scans were processed using the Automatic Lesion Identification (ALI) toolbox [17], which is an elaboration of the popular Unified Segmentation algorithm [18], adapted for use in the damaged brain. The ALI toolbox derives continuous lesion evidence at the voxel level by comparing each participant's scan to a distribution of reference scans acquired on the same scanners from neurologically normal controls. The result is a whole‐brain continuous lesion image in standard Montreal Neurological Institute space. Binary lesion images are thresholded continuous lesion images (threshold = 0.3).

These binary lesion images were then re‐encoded as lesion load variables, representing the proportion of a series of anatomically defined regions (“masks”) that each patient's lesion appears to destroy. We drew the 116 region masks from the first iteration of the Automatic Anatomical Labelling Atlas [19]. The masks all refer to grey matter regions, parcellating both the cortex and subcortical structures into non‐overlapping regions.

### 2.4 Data for Modelling

Our models' predictor (i.e., independent) variables appended the 116 lesion load variables derived from MRI to a further 8 variables per patient. The extra variables convey: (a) time post‐stroke at scan; (b) age at stroke onset; (c) pre‐stroke handedness; (d) sex assigned at birth; (e) whether English was their native language; (f) total lesion volume; and also lesion volume in the (g) left and (h) right hemispheres, respectively. Our models therefore have a total of 124 predictor variables per patient.

We treat the outcome prediction problem as a binary classification, thresholding the language outcome score variables into binary classes. Thresholds in each case are defined by the authors of the CAT [13]. Scores below the threshold fall in the lower 5% of a normative distribution of scores in the same tasks, assigned to a separate sample of 27 neurologically normal controls. Scores in this range are considered to be in the ‘aphasic’ range, whereas scores above the threshold are considered to be in the ‘normal’ range. This is a crude representation of the participants' language outcomes, because it may mask variation (e.g., within the aphasic range) that is both scientifically and clinically meaningful—and that patients might want to know. Nevertheless, this is a natural and well justified starting point for analyses like ours, that aim to measure whether precision‐tuning can generalise out of sample in this domain.

### 2.5 Classification Models

Our binary language outcome variables are often unbalanced, with more participants in one group than the other (see Table 1). So, we attempted to predict these outcomes using a Random Under‐Sampling Boosted (RUS Boosted) classification ensemble [20]. RUS Boosting is specifically designed to cater to classification problems with unbalanced classes. As the name suggests, the method works by randomly excluding some cases from the majority class during training. All of the other hyperparameters of the model were left at their default values, as specified by Matlab 2024a. The default parameters are as follows: ‘NumLearningCycles’ = 100; ‘LearnRate’ = 0.1; MaxNumSplits = 10; ‘MinLeafSize’ = 1; and ‘NumBins’ = 10.

**TABLE 1:** Test set confidence (NPV and PPV) and coverage (the proportions of the samples receiving a classification). All figures are presented as the means and standard deviations across 100 cross‐validation runs; coverage is represented as the proportion of the relevant sample that received a classification. The ‘Total no.’ figures refer to the total number of patients in the sample whose scores are either ‘aphasic’ (third column) or ‘normal’ (final column).

Task	NPV %	% classified	Total no.	PPV %	% classified	Total no.
Fluency	87.8 ± 3.2	15.6 ± 2.4	344	97.3 ± 0.5	37.0 ± 2.8	1012
Comp. (aud)	90.0 ± 5.1	6.2 ± 2.3	422	95.2 ± 1.0	25.6 ± 2.7	914
Comp. (writ)	92.7 ± 2.9	9.2 ± 2.1	526	92.1 ± 1.9	11.3 ± 2.1	822
Repeating	92.6 ± 1.5	38.7 ± 8.3	662	83.2 ± 2.7	25.3 ± 5.7	675
Naming	93.5 ± 2.5	14.5 ± 2.8	475	96.8 ± 1.4	11.5 ± 2.5	864
Scene desc.	94.9 ± 2.2	16.3 ± 4.9	569	88.3 ± 2.4	17.8 ± 3.7	766
Reading	90.5 ± 3.2	11.4 ± 2.7	471	93.9 ± 2.1	14.6 ± 2.1	861
Writing	89.2 ± 2.0	27.5 ± 1.9	376	94.7 ± 1.2	18.6 ± 2.0	929

### 2.6 Model Training, Hyperparameter Tuning and Testing

We used nested cross‐validation to train and test one of these models for each of the language outcomes: that is, 8 models in total. The nesting is necessary because we need to tune two key hyperparameters of each model: thresholds that determine whether we will count a patient as having been either ‘classified with high confidence’, or ‘not classified with high confidence’. Like many other classification models, a RUS Boosted tree actually does more than just assign cases to classes. These models also predict a continuous ‘score’ variable for each class: that is, a total of 2 score variables in the case of binary classification, which is how our current outcome prediction problem is framed. The detailed ranges and relationship between these variables will vary depending on the particular model being employed, but the default behaviour of these models is that cases are assigned to the class with the higher score variable value.

Our tuneable model hyperparameters are thresholds on these score variable values: one threshold for each of the two outcome classes that we consider. We use them to interfere with the models' default behaviour. Rather than assigning a case to the class with the highest score variable value, we only count the classification if the score variable value is higher than our defined threshold for that class. In effect, we are selecting only the most confident subset of the model's classifications. The chance that these classifications are correct—their precision—should be higher for this subset.

In each fold of the nested cross‐validation, we divide the sample into training set (80%), validation set (10%) and testing set (10%). We train a classification ensemble on the training set, then use it to predict outcomes for the validation set. This is the stage at which we search for score variable thresholds that maximise the precision for both classes (i.e., ‘aphasic’ and ‘normal’ range). Then, we use both the trained model and the score thresholds to predict outcomes for the testing set—counting only those classifications whose score variable values surpass our thresholds. Reported results are precision estimates for both the positive prediction (that a patient's outcome will be in the normal range), and the negative prediction (that a patient's outcome will be in the aphasic range). In the results, we refer to these as the Positive Predictive Value (PPV) and the Negative Predictive Value (NPV), respectively.

### 2.7 Analyses 1 and 2

Analysis 1 repeats our procedure 1000 times, using the whole sample, and reports the results. However, since our sample is cross‐sectional, we have no objective data concerning their language skills soon after stroke. Therefore, there is a strong risk that some of the participants whose outcomes are ‘normal’ in any given language domain, might never have suffered aphasic symptoms in that domain. Since clinical stroke populations will presumably only want such predictions if they are suffering from aphasia, this is a potentially dramatic shift in the distribution of our sample, relative to the target population.

Analysis 2 addresses the confound by restricting the predictions in each analysis to patients who reported a relevant, functional impairment at 1 month post‐stroke. Only a subset of patients made these subjective reports, via our in‐house questionnaires, if they remembered their skills in understanding, speaking, reading, and writing. Language ability levels were reported on 7‐point Likert scales. In each case, a score of 7 represented the judgment that the relevant skill was perfectly spared/recovered at 1 month post‐stroke. Patients used a score of 1 if they had no memory of their skills at 1 month post‐stroke—perhaps because they were unconscious at the time, or otherwise too ill to make the judgment. Therefore, we included only patients who scored less than 7 and more than 1 in these analyses.

Analysis 2 is complicated by need to map between subjective self‐report scores and objective outcome scores. Subjective scores for ‘reading’ and ‘writing’ naturally map to our objective outcome scores for the same language skills. The subjective score for ‘understanding’ is taken to describe early symptoms relevant to both of the two ‘comprehension’ outcomes: written and auditory comprehension. And participants were considered to have been initially impaired on all of the four other language tasks—fluency, naming, scene description and reading—if the reported impaired ‘speaking’ soon after stroke.

## 3 Results

### 3.1 Analysis 1a: Training Versus Validation NPV and PPV

**FIGURE 2:**
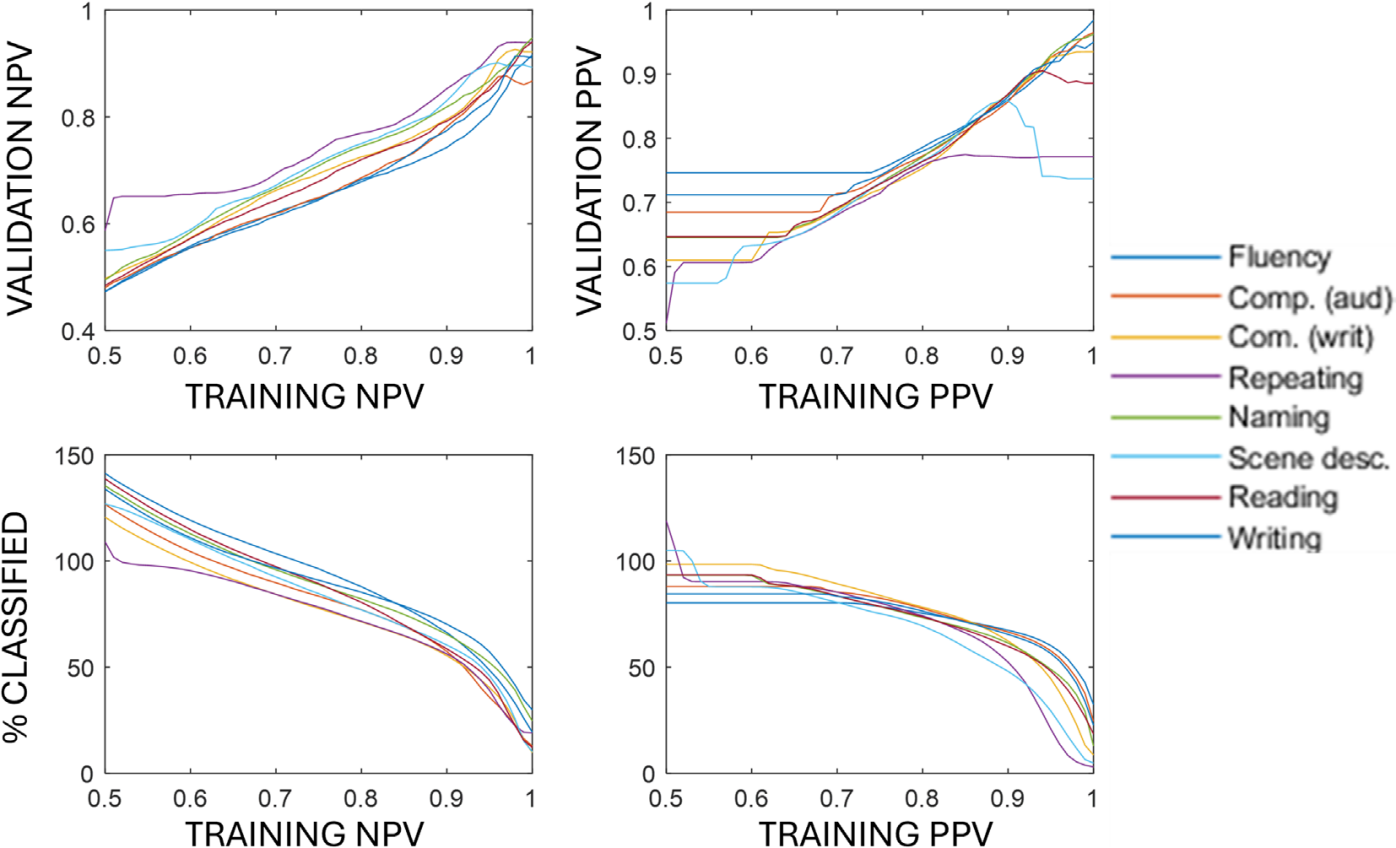
Average validation set classification performance versus training set confidence. For both NPV (top left) and PPV (top right), the validation set values are generally lower than the training set values indicating some absolute loss of performance. Nevertheless, validation set NPV and PPV are both high in the best case. The bottom row shows the proportions of the correct class that receive each type of ‘high confidence’ prediction. Note that when the predictions are made with the most permissive NPV and PPV thresholds, these proportions are often > 100% because these predictions are also being made for a fraction of the sample that compose the other (wrong) class. Notably, the functions relating validation set coverage to training set PPV are convex (bottom row). This suggests that the coverage for these predictions would increase rapidly with only a comparatively minor reduction in threshold precision.

As expected, there is a strong relationship between the within‐sample NPV and PPV thresholds and the same figures when measured in the validation set (Figure 2, top row). However, the relationship is still imperfect; for example, for very high training set thresholds of PPV (> 95%), some loss of PPV is often observed in the validation set.

### 3.2 Analysis 1b: Test Set NPV and PPV

Table 1 reports the NPV and PPV/precision of our confidence‐tuned classifications, as well as the numbers of patients for whom those ‘highly confident’ classifications are made (Figure 1, bottom row). These results illustrate surprisingly high confidence. 6/8 PPV values are > 90%, and 3/8 are > 95%. The lowest PPV values were for the repeating task (83.2%) and the scene description task (88.3%). 6/8 mean NPV values were also > 90%, the exceptions being for the fluency task (87.8%) and the writing task (89.2%). The best result was for the prediction that patients would have fluency skills in the normal range, which was correct 97.3% of the time.

### 3.3 Analysis 2: Test Set NPV and PPV for Patients Reporting Acute Impairment

This analysis is the same as Analysis 1b, except that in this case we restricted the analyses to patients who reported an initial impairment in a language function that the outcome score purports to measure. This restricted sample naturally resulted in a reduction of the numbers of patients for whom a classification is made, primarily because those who reported normal speech soon after stroke were no longer contributing to confident NPV results. However, this made little difference to the out‐of‐sample PPV and NPV results (Table 2). The biggest reduction in confidence was for the NPV for outcomes in the fluency task, which dropped from 87.8% to 82.1%. The biggest improvement was for the NPV for outcomes in the auditory comprehension task, from 90% to 97.9%.

**TABLE 2:** Test set confidence and coverage as in Table 1, but including only patients who reported suffering from a relevant, functional impairment 1‐month post‐stroke.

Task	NPV (%)	No. classified	Total no.	PPV (%)	No. classified	Total no.
Fluency	82.1 ± 9.7	79.0 ± 6.0	138	97.2 ± 0.8	32.8 ± 2.5	393
Comp. (aud)	97.9 ± 4.0	32.9 ± 4.7	130	95.8 ± 2.0	22.5 ± 3.2	275
Comp. (writ)	96.7 ± 2.7	10.4 ± 2.2	171	92.0 ± 3.8	10.1 ± 2.2	240
Repeating	87.0 ± 1.2	18.8 ± 4.5	253	79.0 ± 4.5	23.2 ± 5.6	268
Naming	91.4 ± 3.8	15.5 ± 3.7	187	99.0 ± 1.6	10.4 ± 2.5	334
Scene desc.	93.0 ± 1.8	11.2 ± 3.7	223	86.1 ± 4.9	9.8 ± 3.2	298
Reading	91.4 ± 4.8	18.5 ± 3.4	137	98.3 ± 2.1	12.8 ± 2.3	274
Writing	89.0 ± 2.3	35.2 ± 5.0	120	97.0 ± 1.6	15.0 ± 2.1	294

### 3.4 Model Interpretation

Although our principal aim in this paper was to show that the precision tuning procedure can generalize out of sample, we are naturally also curious as to what is driving their very strong performance.

To capture the importance of predictor variables in our models, we used the standard ‘predictorImportance’ function, as distributed under Matlab 2024a, which calculates importance as the average reduction in class purity when using each predictor to split the data, wherever that predictor is used in the larger classification ensemble. ‘Class purity’ is the proportion of cases assigned to the same class that are really members of the same class; higher purity implies higher accuracy in the classification. Left hemisphere lesion volume was the most important variable in 5/8 tasks, and one of the 10 most important in a further two tasks. This is consistent with the well‐known fact that the left hemisphere plays a dominant role in language function [21]. And the most important variable for the 8th task, repetition, was damage in the left superior temporal lobe, which is a large left hemisphere region that might be a good proxy for left hemisphere volume, and was also a top 10 predictor for every other language outcome. However, damage in this region might also be especially relevant to auditory word repetition because it includes the left temporal gyrus, which is known to support the maintenance of auditory short‐term memory [22].

Another key brain region appeared to be the left middle temporal gyrus (LMTG), which had top 10 importance across 6/8 tasks. The LMTG is involved in the storage and retrieval of semantic memory, and specifically in lexical and syntactic processing, as well as having extensive connectivity to other key language areas such as Broca's area (which has long been claimed to orchestrate speech production) [23]. The exceptions, where LMTG damage was less relevant, were the repeating and reading tasks. Conceivably, this reflects patients' ability to perform these tasks using phonetic rather than semantic processing.

The most consistently significant, non‐brain factor was time post‐stroke at assessment: top 5 for all tasks except for reading (19th). This is consistent with a long history of our and others' prior work, which emphasises the role of this variable in analysing post‐stroke outcomes [4]. To examine this further, we conducted a post hoc analysis on the times post‐stroke at assessment for patients deemed to be ‘predictable with high confidence’ versus ‘not predictable with high confidence’, in each of the 8 tasks. In each case, the groups were defined by the average of classifications made across all 100 repetitions of the cross‐validation for each task: patients assigned to a class more often than not (after applying our thresholding procedure) were placed in the ‘predictable’ group. Using Wilcoxon ranked sum tests, we then compared the times post‐stroke at assessment across the two groups, and as expected, high confidence classifications tended to favour patients assessed later post‐stroke. The differences just missed significance for the auditory comprehension and naming tasks (*Z* = 1.82, *p* = 0.069; and *Z* = 1.79, *p* = 0.075, respectively), but was significant for the other 6 tasks: fluency: *Z* = 2.05, *p* = 0.04; written comprehension: *Z* = 3.04, *p* = 0.002; repeating: *Z* = 2.67, *p* = 0.008; scene description: *Z* = 4.26, *p* < 0.001; reading: *Z* = 4.12, *p* < 0.001; writing: *Z* = 3.81, *p* = 0.002.

Another consistently important factor was bilingualism—which coded whether or not the language of assessment (English) was also the participant's native language. Native English speakers were found to score higher in most CAT tasks in a prior study of a subsample of the current sample [24], so it is no surprise here that non‐native English speakers were generally assigned a lower chance of having ‘recovered’ when assessed. Age at stroke onset was the 2nd most important variable for 2/8 tasks (fluency and reading), and the 13th most important for the auditory comprehension task, but was not in the top 20 for any other task. As expected, participants who were younger at stroke onset were assigned a higher chance of recovering in these tasks: fluency: (*Z* = −2.90, *p* = 0.004; reading: *Z* = 3.16, *p* < 0.001). These results were calculated using the same approach as employed to analyse the effect of time post‐stroke, but this time comparing only the groups predicted to recover, to the groups predicted not to recover, for each task.

Pre‐stroke handedness and sex (assigned at birth) were generally not assigned much importance, with the only marginal exception being for the reading task, where they were the 20th and 21st most important variables, respectively. This suggests that the sex distribution bias in our sample (909 men: 458 women) might not play an important role in our results. However notably, there were significant differences in the proportions of men and women deemed to have ‘highly predictable’ outcomes in 6/8 tasks: see Table 3. There were disproportionately more women in the ‘predictable’ group for the fluency task (*Z* = 2.65, *p* = 0.008), but this was reversed for the other 5 tasks, perhaps suggesting that sex plays a slightly more significant role here than our feature importance analyses suggest.

**TABLE 3:** The proportions of women in the groups whose outcomes were deemed to be either predictable with high confidence or not predictable with high confidence. The table also reports the *p*‐values and *z*‐scores for two proportion *z*‐tests employed to compare whether these proportions were significantly different for each of the 8 outcome scores that we considered.

Task			% women	
	*p*	*z*	Predictable	Not predictable
Fluency	0.008	2.65	39.67	31.43
Comp. Aud	0.018	−2.36	24.03	34.36
Comp. Spk	0.011	−2.54	18.75	34.09
Repeating	0.003	−3.00	23.03	34.83
Naming	0.037	−2.08	25.53	34.28
Scene Desc.	0.010	−2.58	24.00	34.55
Reading	0.755	0.31	34.48	33.19
Writing	0.110	−1.60	26.21	33.95

Indeed, all of these model interpretation results should probably be interpreted with caution, because as yet, we are not sure how to properly account for the way in which our analyses interfere with default model behaviour. For example, the feature importance metrics take no account of our score variable thresholding procedure, so might weight some predictor variables highly even though they play comparatively little role in deciding which patients receive a ‘high confidence’ classification (and vice versa). In other words, we are not sure that our analyses here are as meaningful as they should be, and also not sure how to adjust them: this a focus of ongoing work.

## 4 Discussion

When predicting the outcomes of a large sample of stroke patients, we have shown that, for many language impairments, it is possible to: (a) distinguish ‘more predictable’ from ‘less predictable’ patients; and (b) make high confidence predictions for the more predictable patients, in independent samples. Though likely familiar to many other fields, this is the first application of these methods (as far as we know) to the problem of predicting language outcomes after stroke.

Naturally, at least without new data or methods, our precision‐tuning procedure trades confidence against coverage. As mentioned previously, in the context of binary regression, our classification model (and many others too) will return exactly two ‘score’ variables when presented with a case: one for each of the two classes to which that case might be assigned. The class with the higher score variable value corresponds to the class label that the model will predict. Our precision tuning procedure interferes with this default behaviour, by specifying thresholds for those score variables. We only ‘count’ a classification as having been made, if the score variable value associated with a class is above the threshold for that class. As those thresholds increase, more cases will receive no classification at all. This is why coverage tends to decrease. But ever‐higher thresholds also restrict the model to cases where it is most confident in its classification. In turn, this suggests that the model is more likely to be correct for those cases. This is not guaranteed, but our results show that it does in fact happen: that we gain higher precision, albeit at the cost of reduced coverage.

To make the point that precision tuning can generalise out of sample, in this domain, we have tuned our models to maximise their precision, regardless of the cost in terms of coverage. Future variants of our approach might well benefit from taking a more balanced approach: that is, making predictions for more of a sample, by lowering our score variable thresholds and thus accepting a lower level of required precision. Notably, our results suggest that the coverage of our predictions (i.e., the number of patients who receive a ‘high confidence’ prediction) will increase rapidly with small reductions in target precision. This is implied by the convex curves, relating coverage to required precision, in the lower panels of Figure 2.

Quite what the optimal balance is between coverage and confidence will be a matter for clinical judgement—and might vary both depending on the direction of the prediction (normal or impaired) or indeed even the patients themselves. For example, some patients might prefer not to know their prognosis at all if the news is bad, no matter how high its confidence. And some might want to hear potentially good news even if its confidence was much lower than we have targeted here. To respect those variable preferences, the calibration of our confidence—its reliability and validity—might be more important than its absolute level. We make no assumptions about what levels of confidence might be appropriate for either side of this prediction in clinical practice. Our aim here has simply been to direct attention to this as‐yet under‐studied dimension of the problem by showing that high confidence predictions can in principle be made.

Confidence in that calibration should be higher as the sample used to measure it grows larger, and our PLORAS sample is large by the standards of the field. However, even in large samples, selection biases can make *distribution shift* a likely barrier to clinical exploitation. Since our confidence measures are made over the samples we have, they could well change if the distributions of clinical populations are different in important respects. Analysis 2 was motivated by one likely cause of distribution shift in Analysis 1: the inclusion of patients who did not report initial impairment of the language functions whose outcomes we were predicting. Since we presume that clinical populations will be restricted to those suffering from a relevant, acute impairment, Analysis 2 implemented that restriction, and its results suggest that our confidence‐tuning method is robust to that distribution shift.

Despite the encouraging results, Analysis 2 had a number of limitations because our measure of acute impairment is (a) subjective; (b) retrospective given that it depends on patients' memories of their past symptoms; (c) imperfectly matched to the objective outcome scores (as discussed in the Methods); and (d) was only collected for a subset of the sample. Ongoing work aims to address these limitations. There are also other likely distribution shifts to consider. For example, 73.7% of our PLORAS patients were under 65 at stroke onset, whereas ~70% of patients in the wider stroke survivor population were older than 65 when their strokes occurred. From a separate analysis of subsets of our own study sample, we know that age at stroke onset is relevant to our language scores (Roberts et al., in submission). Other biases are likely driven by the exclusion of patients suffering from concurrent, serious neurological disorders (such as Alzheimer's or Parkinson's disease) and the predominance of patients who were tested many years post‐stroke. Such biases are probably inevitable, particularly since our sample only included patients who were able to travel to our assessment facilities in central London.

These statistical barriers to clinical translation are accompanied by technical barriers—mostly concerning the reliance of our current analyses on lesion data derived from research quality MRI, acquired during the chronic phase post‐stroke. Clinically routine MRI is typically acquired at lower resolutions and with more variable contrasts and fields of view. Lesion segmentation is already difficult in research‐quality brain images [25]: clinical images pose a more formidable challenge. And even if that technical challenge can be met, the underlying lesion ‘signal’ in brain scans acquired acutely might be obscured by oedema [26]. Work to surmount these technical and statistical challenges is ongoing.

Finally, our analysis is also somewhat divorced from its practical context because our representation of ‘recovery’, as a binary variable, is likely too simplistic. In the past, we and others have used richer and more continuous representations of recovery, and predicted them with regression models instead [4, 5, 27, 28, 29]. These more continuous representations are attractive because they can capture variation that is both clinically and scientifically significant, such as recovery from more severe aphasia to less severe aphasia. In this work, we have effectively ignored that variation for pragmatic reasons—because a focus on the binary ‘aphasic/recovered’ distinction is the simplest and best justified starting point, when the goal is to measure whether precision tuned predictions can generalise out of sample, in this domain. But our methods here could well be extended to capture more (or all) of that more detailed variation. This could be done by varying the cut‐off thresholds that we impose, as others have done in related work, such as: (a) distinguishing ‘severe impairment’ from ‘mild or moderate’ impairment [30]; or (b) distinguishing ‘excellent recovery’ from other types of recovery [31]. Indeed, both of those adjusted cut‐off thresholds could also be used together with the threshold that we have employed (‘aphasic versus recovered’), and potentially many others as well. By training multiple pairs of models to make high precision decisions for multiple binary classification thresholds, our precision tuning procedure can naturally be extended to capture the dynamic range of the outcome scores at any level of precision that is deemed to be scientifically or clinically useful.

Despite their limitations, our current results demonstrate that it might be fruitful to take a different perspective on this problem, which has challenged so many researchers for so long. Over more than a decade, we and others have tackled it by acquiring ever larger samples and attempting to derive ever better predictive results across those whole samples [4, 5, 12, 24, 27, 28, 30, 32, 33, 34]. Given the difficulty of the problem of providing high confidence predictions for every patient, we suggest that, as a first step, we should focus on solving the problem incrementally—delivering what clinical benefit we can now (or soon), while working to expand the pool of patients for whom high confidence prognoses can be made.

## Author Contributions

T.M.H.H., C.J.P., and H.B. contributed to the conception and design of the study. T.M.H.H., C.J.P., R.M.B., and A.P.L. contributed to the acquisition and analysis of the data. T.M.H.H. and C.J.P. contributed to drafting the text or preparing the figures.

## Disclosure

Open Access: The authors have applied a CC BY public copyright licence to any Author Accepted Manuscript version arising from this submission.

## Conflicts of Interest

The authors declare no conflicts of interest.

## Data Availability

Patient data are available on the conclusion of a data sharing agreement with University College London. Interested researchers should contact C.J.P. in the first instance. Software developed to run these analyses can be accessed at https://github.com/tmhhopegit/precision_prognoses.
